# It’s Never Too Early or Too Late—End the Epidemic of Alzheimer’s by Preventing or Reversing Causation From Pre-birth to Death

**DOI:** 10.3389/fnagi.2018.00205

**Published:** 2018-07-12

**Authors:** Clement L. Trempe, Thomas J. Lewis

**Affiliations:** ^1^Independent Clinician, Nahant, MA, United States; ^2^RealHealth Clinics, Jefferson City, TN, United States

**Keywords:** Alzheimer’s, infection, systemic, diabetes, congenital, immunity, prevention, inflammation

## Abstract

The path to sporadic Alzheimer’s is a tragic journey beginning prior to birth and ending in the most dreaded disease of society. Along the disease path are a myriad of clues that portend AD, many of which are complaints of seemingly unrelated conditions from chronic migraines, mood disorders, eye diseases, metabolic syndromes, periodontal diseases, hormonal and autoimmune diseases. Properly treating, not just managing, these diseases, prior to onset of dementia, may significantly reduce dementia incidences. Current high levels of health complaints reflect a state of generalized poor health and compromised immunity. During the mid-Victorian era, people were long-lived yet healthy, suffering from chronic diseases at one tenth the rate of peoples today. It’s our poor health, at any age that increases susceptibility to chronic diseases and Alzheimer’s. Infection is involved in many cases of Alzheimer’s and other neurodegenerative diseases but is also implicated in many chronic conditions. Scientists looking for causation recognize that Alzheimer’ is multifactorial and systemic—not “brain only.” To slow, stop and reverse the AD epidemic, identification and reversal of causal factors must occur across the entire life spectrum of humans. This approach simply gives consideration to enhancing immune status of our bodies and brain, and controlling inflammation and infection, throughout the entire age spectrum. Infection is a causal factor, but the root cause is multi-factorial and immune health related. Pasteur stated it best when acknowledging the work of Bernard in 19th Century France, “The seed is nothing, the soil is everything.”

## Introduction: Immunity and Infectious Diseases

Historically, infectious diseases were the cause of morbidity and mortality. Infectious disease arguably continues to be the major driver of morbidity and mortality however this connection is largely ignored because of the occult nature of many of the causative agents and the cryptic cause and effect between organism and disease (Cochran et al., 2000). Dr. Paul Ewald explains how the concept of evolutionary fitness actually points to infection as being the major cause of disease in modern society (Cochran et al., 2000). Evolutionary fitness means the evolutionary success of an organism relative to competing organism. Genetic traits that may be unfavorable to an organism’s survival or reproduction do not persist in the gene pool for very long. Natural selection weeds them out and any inherited disease or trait that has a serious impact on fitness must fade over time. Therefore, in considering common chronic conditions with severe health consequences, we may presume a non-genetic cause. According to Ewald (2002), “When diseases have been present in human populations for many generations and still have a substantial negative impact on people’s fitness, they are likely to have infectious causes.”

Immune system vitality may be the most important risk factor in any chronic disease including Alzheimer’s. The World Health Organization in “Risk Factors of Communicable Diseases” (World Health Organization, 2011) states, “Apart from symbiotic coexistence of human with micro-organisms, disease causing organisms breed in man-made unhygienic conditions of air water and soil. *People with low immunity*, *weak*, and living in unhygienic conditions are at greater risk for contracting the infections from surroundings.” This model of disease fits equally well with Alzheimer’s and other chronic diseases but has been limited because source of the infection is less obvious and diagnosis is not frequently enough made or considered.

Chronic inflammation is considered a cause of chronic disease, including Alzheimer’s (Rogers, 1995; Holmes et al., 2009; Kawai et al., 2018). As defined by Opie (1929) “Inflammation may be defined as the process by which cells and serum accumulate about an injurious agent and tend to remove or destroy it.” Chronic inflammation continues to be blamed for tissue damage but this complex cascade, stimulated by internal and external mediators, results in the release of danger signals that promote immune responses to antigens (Rock and Kono, 2008). Chronic, occult infection is a significant stimulator of chronic inflammation (Beatty et al., 1994). Host-pathogen interactions, defined as the importance of the host’s susceptibility for a microbe’s virulence, must be considered and this interrelationship is not straightforward. Casadevall and Pirofski (1999) proposed six different classes of host-pathogen interactions that helps explain how the relationship between infection, inflammation, immune health and disease, although apparent, may not always yield statistical certainty.

Rivas et al. (2017) explain that the current research paradigm is reductionist yet biological systems combine their limited elements, creating complex structures and solutions involving infectious diseases. The network theory of aging, and particular, inflamm-aging is defined as, “a global reduction in the capacity to cope with a variety of stressors and a concomitant progressive increase in proinflammatory status,” contributes to a more non-reductionist assessment of individualized health by measuring “many” rather than one or a few (Franceschi et al., 2000). The construct of inflamm-aging is a measure of immune system activity against chronic insult. Any chronic disease, then, is potentially a measure of the stress on the biological system and its ability, or lack thereof, to cope.

**Figure 1:**
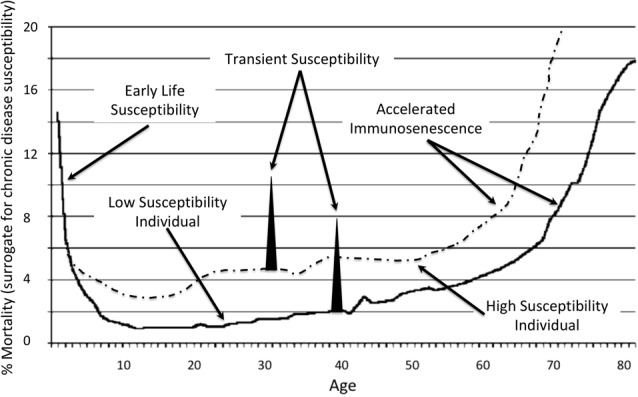
Representation of susceptibility to chronic diseases and Alzheimer’s across the age spectrum (House, 2018).

Chronic disease incidences, including Alzheimer’s, increases with older age and are linked to immunosenescence (Solana et al., 2012). Numerous studies show that the pathology of Alzheimer’s disease is present decades before a clinical diagnosis of dementia can be made (Mortimer et al., 2005). Predisposition to Alzheimer’s, therefore, is established prior to the acceleration of immunosenescence that starts around age 65 (Figure 1). The curve also represents the vulnerability to disease due to an immature immune system during the ages 0–5. It is during this time that the antecedents of Alzheimer’s and other chronic diseases, specifically occult infections, may opportunistically infiltrate such vulnerable hosts only to express as disease across the spectrum of time and lead to the a significant upswing in Alzheimer’s. A comparison of a healthy and unhealthy person is also represented as are transient susceptibilities that may include acute health or sudden life changing events that adversely impact health.

There are numerous occult ectopic obligate intracellular pathogens linked to Alzheimer’s, including *Chlamydophila pneumoniae* (CP; Balin et al., 1998), *Borrelia burgdorferi* (BB; Marquand and Muller, 1997), gram-negative anerobic bacteria of the oral cavity (Riviere et al., 2002), h-pylori (Malaguarnera et al., 2004), rickettsias (Morrison et al., 1991) and *Toxoplasma gondii* (Jung et al., 2012). Many infectious species are ubiquitous within both human and animal populations. CP expresses seroprevalence rates of over 50% among adults in the United States (Blasi et al., 2009). In some vulnerable individual, CP migrates to the brain and contributes to neurodegeneration (Gérard et al., 2006).

The connection between immune system health and infection extends back to the research of Bernard and Pasteur (Garko, 2012). Modern studies at MIT demonstrate a strong correlation between immunity and future Alzheimer’s (Dougherty, 2015). The CDC, in an article on neglected parasitic infections explain how, of the 60 million Americans infected with the Toxoplasma parasite, only a small proportion of the susceptible experience severe health consequences (Wong and Remington, 1994; Chaudhry et al., 2014; Jones et al., 2014). Higher prevalence of *T. gondii* in patients with AD compared to controls shows an association between organism and disease (Schulz et al., 2007; Rashno et al., 2016, 2017). An estimated 18% of the United States are seropositive and worldwide, an estimated one third of the population are infected (Dalimi and Abdoli, 2012). Chronic infection is reported to reactivate toxoplasmosis (Montoya and Liesenfeld, 2004). For the individuals infected, any susceptibility factor illustrated in Figure 1 may contribute to toxoplasmosis proliferation and potential exacerbation of Alzheimer’s.

The CDC indicates that few infected with toxoplasmosis have symptoms. Yet, according to Kaiser Family Foundation, the highest percentage of medical spending occurs for ill-defined conditions (Peterson-Kaiser Health System Tracker, 2018). Toxoplasmosis and other occult organism likely contribute to these conditions and associated high cost of care. Stratton and Wheldon (2006) explains the life cycle of CP and its treatment when discovered associated with multiple sclerosis and heart diseases. Dr. Wheldon contributed the following list of disease presentations he associated with CP in his clinical practice over 20 years: cardiac conduction defects; effusive pericarditis with tamponade; chronic obstructive airways disease; multiple sclerosis; Alzheimer’s disease; chronic fatigue syndrome; encephalitis; retinal vasculitis; macular degeneration; progressive presbyopia; Crohn’s disease; new onset adult asthma; and schizophrenia (hebephrenia). A similar list associated with Toxoplasmosis may be established with more research.

Lyme disease, caused by vector spirochetes, is a well-recognized chronic disease precipitated from BB and co-infections. Miklossy hypothesized on a link between BB and Alzheimer’s in 1994 and proved a causal relationship (Miklossy, 1994, 2011). The CDC recently revised its estimate of 30,000 new cases per year in 2014 to 300,000+ in 2017 in the United States Kris Kristofferson was diagnosed with AD yet, after a discovery of Lyme disease, the public message was, “Kristofferson struggled with memory problems in recent years and was told he had Alzheimer’s disease, but it appears he was misdiagnosed and all along has actually been suffering from the tick-borne illness Lyme disease” (Marcus, 2016). The association and causation between infection and Alzheimer’s continues largely disregarded.

In “Overview of Lyme Disease: A Critique of an Ignored Pandemic,” Dr. Ken Stoller suggests that 20% of the human population is infected and may well explain diseases of unknown origin (Stoller, 2015). In a recently completed investigation of a cohort of 70 functioning but unhealthy manufacturing company workers in central Indiana, we evaluated 41 for stealth infection, including Lyme disease, based on reported lifestyle, symptoms, a broad array of inflammatory biomarkers, and serology testing. Of the 41, 40 were positive for at least one occult organism and most had two or more, 28 were found to have positive IGG titers for CP; 12 were positive for *Toxoplasma Gondii*; 18 were positive for at least one IGG band for Lyme; one was positive for Q Fever and one was positive for Rickettsia Typhii (Lewis et al., submitted). Presuming that the 30 untested individuals were negative for Lyme, these data support the estimated population infectious burden suggested by Stoller.

## Pre-birth Susceptibility to Future AD

Congenital infections affect the unborn fetus or newborn infant. Conventionally, they are thought to be caused by viruses that may be picked up by the baby at any time during the pregnancy. Even if the mother is known to have a viral illness during her pregnancy, her immune system may prevent the virus from infecting the fetus or newborn infant. However, shortly after birth for mothers that do not breast feed or shortly after stopping breast feeding, the newborn’s immune system function drops precipitously and then slowly strengthens from ages 0–5, Figure 1.

Periodontal bacteria is a significant contributor to congenital infection. Offenbacher et al. (1996) were the first to report a relationship between maternal periodontal disease and delivery of a preterm infant. Global incidence of preterm birth is around 9.6% with regional disparities from 12% to 13% in USA, 5% to 9% in Europe, and 18% in Africa. According to “Born Too Soon,” the United States ranked 131st out of 184 in preterm births (Blencowe et al., 2013). Full term stillbirth is also caused by bacteria including Fusobacterium nucleatum (Han et al., 2010). The placenta is an immuno-suppressed organ compared to other organs like the liver and the spleen which makes it easy for the bacteria to colonize there. Intrauterine infection is considered a leading cause of early preterm birth and data from clinical and experimental studies suggest that infection accounts for upward of 40% of these high-risk preterm deliveries (Kravetz and Federman, 2005; Kemp et al., 2017).

Determining a statistical association between periodontal infection induced premature births and dementias is challenging because of the 70+ year gap between birth and disease. In “Birth in a High Infant Mortality State: Race and Risk of Dementia,” higher rates of dementia later in life was statistically connected to higher incidences of infant mortality in racial cohorts (Gilsanz et al., 2017). In a cohort of elderly individuals, blacks born in states with the highest levels of race specific infant mortality rates had 40% increased risk of dementia compared to national averages. Early life conditions may contribute to racial inequalities in dementia incidence according to the authors. Since infection accounts for a preponderance of preterm deliveries, one can surmise that the excess of dementia is tied back to either a failure to thrive at birth (poor immune status), chronic infection acquired during pregnancy, or a combination of these adverse circumstances, all of which add risk for future Alzheimer’s upon immunosenescence (Jeffcoat et al., 2002), Figure 1.

Non-periodontal infections are connected with both adverse pregnancy outcomes and dementias. Cytomegalovirus (CMV) infection in pregnancy causes adverse clinical outcomes and the rate of transmission *in utero* was reported at roughly 33% (Stagno et al., 1987). CMV is present in a very high proportion of brains from vascular dementia patients (Lin et al., 2002). It has also been implicated as a risk factor for Alzheimer’s (Barnes et al., 2014). Besides the inflammatory response elicited by infection, CMV may also drive immunosenescence and thus make an individual more susceptible to Alzheimer’s as a result of the infection or a co-infection acquired during any time in lifespan, Figure 1. According to Dow, “In immune competent individuals, infection with CMV is usually asymptomatic, even in neonates, but once established, its containment becomes a priority for the immune system, which is unable to completely eliminate it (Dow, 2015). However, even healthy immune competent people may display symptoms of CMV infection more often than previously appreciated, sometimes even with serious consequences and with age implicated as a risk factor. The consequences to the immune system for maintaining this constant CMV vigilance may be severe. Reports on the very young and the very old show that CMV infection results in similar alterations to CD8+ T cell subset surface phenotypes. This has given rise to the concept that what are apparent age-associated changes could rather be due to age-associated increases in prevalence of CMV infection.” This phenomenon may be driven by other pathogens as well with susceptibility and disease incident being explained by the proposed different classes of host-pathogen interactions (Casadevall and Pirofski, 1999).

## Early Life Signs and Susceptibility to Future AD

In the United States, the peak age at diagnosis for Type 1 diabetes (T1D) is 10–14 (Maahs et al., 2010). Genetics may play a role, but infection is strongly associated with the condition. T1D follows viral infections such as mumps, rubella, CMV, measles, influenza, encephalitis, polio, or Epstein-Barr virus. Viruses play an important role in the pathogenesis of T1D by inducing or accelerating the beta cell destruction process (Christen et al., 2012). Bacteria are also T1D associated agents. CP was found in 46.5% of young patients with T1D compared to 10.5% of non-diabetic controls (Rizzo et al., 2012). Additionally, CP antibody positivity was significantly more common in patients in poor metabolic control (HbA1c >9%) vs. patients in good metabolic control (HbA1c <7%) and dysfunction of pancreatic beta cells (Rodriguez et al., 2015). Older patients with T1D have more than an 80% increased risk for dementia compared with those without diabetes and the risk is even high when T2D subjects are excluded (Whitmer et al., 2015). Thus T1D, and more importantly the potential immune dysfunction and infection associated with the disease, are indicators of future AD.

## Young Life Signs and Susceptibility to Future AD

More than 10% of all primary care office visits are depression- or mood-related (Stafford et al., 2000; Olfson et al., 2014). The research community knows that depression and Alzheimer’s disease are linked and its now clear that depression is a risk factor, not just a symptom, of AD (Dantzer et al., 2008; Wilson et al., 2010) A 17-year prospective study from the Framingham cohort demonstrates that older adults with depressive symptoms are at a 50% increased risk of developing Alzheimer’s disease (Saczynski et al., 2010). In another study, the authors showed an incident AD group compared to those without AD reported a barely perceptible increase in depressive symptoms during 6–7 years of observation before the diagnosis and no change during 2–3 years of observation after the diagnosis (Wilson et al., 2010). According to Caraci et al. (2010) the molecular mechanisms and cascades that underlie the pathogenesis of major depression, such as chronic inflammation and hyperactivation of the hypothalamic-pituitary-adrenal (HPA) axis, are also involved in the pathogenesis of AD (Caraci et al., 2010). Johnson et al. (2015) found that the signs of depression begin well before the symptoms of dementia begin manifesting. They believe that there are a core group of mood and depression symptoms, easily measureable in populations, that may enable medical professionals to differentiate between people at risk of developing dementia and normally aging individuals.

In “Latent Toxoplasmosis and Humans,” the authors review the impact of toxoplasmosis in the etiology of different mental disorders including schizophrenia and depressive disorders, obsessive-compulsive disorder, Alzheimer’s and Parkinson’s disease, epilepsy, headache and migraine, mental retardation, suicidal tendencies and intelligence (Dalimi and Abdoli, 2012). *Toxoplasma gondii* impairs memory in infected seniors without diagnosis of neurodegenerative disease. In a study of executive function and memory, subjects positive for Toxo showed memory performance reduction of about 35% compared to the toxo negative group and this group also reported a decreased quality of life compared to those not infected (Gajewski et al., 2014). In our own study cohort of 70 individuals, 40 were tested for IgG antibodies for toxoplasmosis and 12 were positive (Lewis et al., submitted).

The causal agent of Lyme is reported in mood disorder of young and middle-aged people (Fallon et al., 1993; Dersch et al., 2015). Determining causation from infection in mood disorders and suicidal tendencies suffers from crypticity. Garakani and Mitton (2015) state, “The patient’s panic attacks resolved after he was discharged and then, months later, treated with long-term antibiotics for suspected “chronic Lyme Disease” despite having negative Lyme titers” (Garakani and Mitton, 2015). A significant feature of post-treatment Lyme disease syndrome is depression (Rebman et al., 2017). Miklossy has provided adequate analysis to convince the most stubborn opponent of the relationship between BB and Alzheimer’s (Miklossy, 1994, 2011; Miklossy et al., 2004). Depression may be a clue as to the same infectious process and since depression generally precedes Alzheimer’s, treatments may be more effective.

## Young to Middle Life Signs and Susceptibility to Future AD

The Rotterdam study revealed an increased risk of dementia and AD in patients with type 2 diabetes (T2D; Ott et al., 1999). Those with T2D are at 50% greater risk of developing a neurodegenerative condition compared to healthy contemporaries (Mayeda et al., 2015). Insulin resistance early in life may enhance the risk of developing neurodegeneration (Luciano et al., 2015). Insulin resistance is due in part to infection, as first described in 1943 (Greene and Keohen, 1943). The article published in JAMA titled, “Insulin resistance due to infection in diabetes mellitus in man,” has not been widely cited. A modern version concludes that pathogen burden has the strongest association with insulin resistance among all the risk factors considered (Fernández-Real et al., 2006). For example, HSV-2 titer was negatively associated with insulin sensitivity even after factoring for inflammation. The relationship was strengthened further for subjects that were seropositive for CP and Enteroviruses. People with T2D are more prone to be on the “high susceptibility individual” curve of Figure 1.

## Middle Life Signs and Susceptibility to Future AD

The eye is a canary for the brain because the retina is an embryonical outcropping of the brain (Dowling, 1987). Thus, anatomically and developmentally, the retina is an extension of the CNS; it consists of retinal ganglion cells, the axons of which form the optic nerve, whose fibers are, in effect, CNS axons. The eye has unique physical structures and a local array of surface molecules and cytokines, and is host to specialized immune responses similar to those in the brain and spinal cord. Several well-defined neurodegenerative conditions that affect the brain have manifestations in the eye, and ocular symptoms often precede CNS diagnoses by up to 20 years (London et al., 2013). Various eye-specific pathologies share characteristics of other CNS pathologies, including Alzheimer’s disease and glaucoma (McKinnon, 2003).

Immunoglobulin serology for CP is significantly higher in patients with glaucoma compared to controls (Yuki et al., 2010). Other infections noted in association with glaucoma include: *Helicobacter pylori* (Galloway et al., 2003), *Bartonella henselae* (Gray et al., 2004), HIV (Nash and Lindquist, 1992), CMV (Sekhsaria et al., 1992), Toxoplasmosis (Sheets et al., 2009), Tuberculosis (Egbagbe and Omoti, 2008), and Lyme disease (Zaidman, 1997). The eye is susceptible to inflammatory diseases through a delicate balance with survival coined “ocular immune privilege.” According to Streilein, “Not surprisingly, inflammation, if it occurs within the eye, is a profound threat to vision” (Hazlett and Stein-Streilein, 2012). Immune privilege and easy accessibility provides an explanation why neurodegeneration presents in the eye long before the brain in many cases and offers a low-cost early or pre-disease screening tool and link to treatable associations to future AD.

Macular degeneration is both a vascular and neurodegenerative disorder easily observed with simple yet elegant optometric instruments. Studies titled, “Age-related macular degeneration (AMD): Alzheimer’s disease in the eye?” (Kaarniranta et al., 2011) and “Parallel findings in age-related macular degeneration and Alzheimer’s disease,” (Ohno-Matsui, 2011) provide examples of what early neurodegenerative disease diagnosis could be. The less studied common denominator between macular disease and neurodegenerative diseases is infection. CP is a documented actor in age-related macular degeneration (Haas et al., 2009). Our own clinical evaluations, being compiled for publication, show seropositive CP and other occult infections in significant percentages of clinical AMD cases.

## Infection, Inflammation and Alzheimer’s “Hallmarks”

Balin et al. (1998) links CP to Alzheimer’s. Subsequently they have published dozens of articles on this topic including, “Proof of Concept Studies of Chlamydia Pneumoniae Infection as a Trigger for Late-onset Alzheimer Disease” (Balin, 2017). A Harvard-led team was the first to definitely show that the beta amyloid hallmark of AD is actually has antimicrobial properties (Soscia et al., 2010). Ravnskov and McCully explained that microvascular hypoxia is initiated by the sequestration of infectious remnants by LDL particles and this process is also noted in Alzheimer’s (Bailey et al., 2004; Ravnskov and McCully, 2009). Tau hyperphosphorylation is also noted in microvascular disease-associated neurodegenerative diseases (Castillo-Carranza et al., 2017). Hibernating animals also hyperphosphorylate tau and the common denominator between Alzheimer’s and hibernation may be hypoxia and, like with beta amyloid, the modified tau serves to protect the brain (Su et al., 2008). Corriveau et al. (2016) explain the significance of vascular contributions to cognitive impairment and dementia. Miklossy et al. (2006) noted increase in inflammation, beta-amyloid precursor protein, and hyperphosphorylated tau production induced by spirochetes or lipopolysaccharide. Balin (2017) delved into the link between the pathogens, inflammation, Abeta 1–42 production, regulation of gene transcripts, and protein expression. The conclusions regarding gene transcripts and protein expression are supported by studies of infectogenomics (Kellam and Weiss, 2006; Nibali et al., 2014). No other single explanation, other than infection, supports all the observed pathologies in Alzheimer’s.

## Conclusion

Sub-optimal health and chronic inflammation predisposes individuals to opportunistic infections and a myriad of diseases throughout the continuum of life provide information about risk and susceptibility to future Alzheimer’s disease. Poor health status, periodontal diseases, congenital infections, depression, diabetes, eye diseases, to name a few, provide a roadmap of progression towards neurodegenerative disorders. Fundamental to the onset and propagation of all these diseases is immune system immaturity, dysfunction and senescence that facilitate proliferation of opportunistic infection. Clinically, medicine must change its paradigm of managing disease symptoms and adopt a proactive approach of testing for immune health status, by way of biomarkers of inflammation, to identify those at risk of future Alzheimer’s and other chronic diseases. Next, each at-risk subject must be provided guidance on how to improve their health status. In addition, those with compromised immune status must also be tested for infection, and if found, treated until the burden is lowered or eradicated. This program must be conducted across the age spectrum for people showing signs and symptoms of any disease associated with Alzheimer’s in later life. This approach could conceivably delay Alzheimer’s onset by 5 years which could reduce number of incidences in half. When clinicians recognize association and causation between earlier chronic conditions and Alzheimer’s and focus on mitigation rather than management, a 50% or greater reduction in the disease becomes realistic.

## Author Contributions

TL and CT co-authored this work. The inspiration for this article is the clinical work of CT. TL is responsible for the research and assembly of the document.

## Conflict of Interest Statement

The authors declare that the research was conducted in the absence of any commercial or financial relationships that could be construed as a potential conflict of interest.

## References

[B1] Bailey T. L. Rivara C. B. Rocher A. B. Hof P. R. (2004). The nature and effects of cortical microvascular pathology in aging and Alzheimer’s disease. Neurol. Res. 26, 573–578. 10.1179/016164104225016272 15265277

[B2] Balin B. J. (2017). Proof of concept studies of chlamydia pneumoniae infection as a trigger for late-onset Alzheimer disease. Neurodegener. Dis. 17:243 Available online at: https://www.karger.com/Journal/Home/229093

[B3] Balin B. J. Gérard H. C. Arking E. J. Appelt D. M. Branigan P. J. Abrams J. T.. (1998). Identification and localization of chlamydia pneumoniae in the Alzheimer’s brain. Med. Microbiol. Immunol. 187, 23–42. 10.1007/s004300050071 9749980

[B4] Barnes L. L. Capuano A. W. Aiello A. E. Turner A. D. Robert Yolken H. Torrey E. F.. (2014). Cytomegalovirus infection and risk of Alzheimer disease in older black and white individuals. J. Infect. Dis. 211, 230–237. 10.1093/infdis/jiu437 25108028 PMC4326304

[B5] Beatty W. L. Byrne G. I. Morrison R. P. (1994). Repeated and persistent infection with *Chlamydia* and the development of chronic inflammation and disease. Trends Microbiol. 2, 94–98. 10.1016/0966-842x(94)90542-8 8156277

[B6] Blasi F. Tarsia P. Aliberti S. (2009). Chlamydophila pneumoniae. Clin. Microbiol. Infect. 15, 29–35. 10.1111/j.1469-0691.2008.02130.x 19220337

[B7] Blencowe H. Cousens S. Chou D. Oestergaard M. Say L. Moller A. B.. (2013). Born too soon: the global epidemiology of 15 million preterm births. Reprod. Health 10:S2. 10.1186/1742-4755-10-S1-S2 24625129 PMC3828585

[B8] Caraci F. Copani A. Nicoletti F. Drago F. (2010). Depression and Alzheimer’s disease: neurobiological links and common pharmacological targets. Eur. J. Pharmacol. 626, 64–71. 10.1016/j.ejphar.2009.10.022 19837057

[B9] Casadevall A. Pirofski L.-A. (1999). Host-pathogen interactions: redefining the basic concepts of virulence and pathogenicity. Infect. Immun. 67, 3703–3713. 10417127 10.1128/iai.67.8.3703-3713.1999 PMC96643

[B10] Castillo-Carranza D. L. Nilson A. N. Van Skike C. E. Jahrling J. B. Patel K. Garach P.. (2017). Cerebral microvascular accumulation of tau oligomers in Alzheimer’s disease and related tauopathies. Aging Dis. 8, 257–266. 10.14336/AD.2017.0112 28580182 PMC5440106

[B11] Chaudhry S. A. Gad N. Koren G. (2014). Toxoplasmosis and pregnancy. Can. Fam. Physician 60, 334–336. 24733322 PMC4046541

[B12] Christen U. Bender C. von Herrath M. G. (2012). Infection as a cause of type 1 diabetes? Curr. Opin. Rheumatol. 24, 417–423. 10.1097/BOR.0b013e3283533719 22504578 PMC4828240

[B13] Cochran G. M. Ewald P. W. Cochran K. D. (2000). Infectious causation of disease: an evolutionary perspective. Perspect. Biol. Med. 43, 406–448. 10.1353/pbm.2000.0016 10893730

[B14] Corriveau R. A. Bosetti F. Emr M. Gladman J. T. Koenig J. I. Moy C. S.. (2016). The science of vascular contributions to cognitive impairment and dementia (VCID): a framework for advancing research priorities in the cerebrovascular biology of cognitive decline. Cell. Mol. Neurobiol. 36, 281–288. 10.1007/s10571-016-0334-7 27095366 PMC4859348

[B15] Dalimi A. Abdoli A. (2012). Latent toxoplasmosis and human. Iran. J. Parasitol. 7, 1–17. 23133466 PMC3488815

[B16] Dantzer R. O’Connor J. C. Freund G. G. Johnson R. W. Kelley K. W. (2008). From inflammation to sickness and depression: when the immune system subjugates the brain. Nat. Rev. Neurosci. 9, 46–56. 10.1038/nrn2297 18073775 PMC2919277

[B17] Dersch R. Sarnes A. A. Maul M. Hottenrott T. Baumgartner A. Rauer S.. (2015). Quality of life, fatigue, depression and cognitive impairment in Lyme neuroborreliosis. J. Neurol. 262, 2572–2577. 10.1007/s00415-015-7891-4 26410742

[B18] Dougherty E. (2015). Understanding the aging brain: new research from li-huei tsai’s lab suggests the immune system may play a role in Alzheimer’s disease. Brain and Cognitive Sciences. Available online at: https://bcs.mit.edu/news-events/news/understanding-aging-brain-new-research-li-huei-tsai’s-lab-suggests-immune-system [Accessed May on 27, 2018].

[B19] Dow C. (2015). CMV driven immunoscenescence and Alzheimer’s disease. J. Neuroinfect. Dis. 6:195 10.4172/2314-7326.1000195

[B20] Dowling J. E. (1987). The Retina: An Approachable Part of the Brain. Cambridge, MA: Harvard University Press.

[B21] Egbagbe E. E. Omoti A. E. (2008). Ocular disorders in adult patients with tuberculosis in a tertiary care hospital in Nigeria. Middle East Afr. J. Ophthalmol. 15, 73–76. 10.4103/0974-9233.51996 21346841 PMC3038112

[B22] Ewald P. W. (2002). Plague Time: The New Germ Theory of Disease. Norwell, MA: Anchor Press.

[B23] Fallon B. A. Nields J. A. Parsons B. Liebowitz M. R. Klein D. F. (1993). Psychiatric manifestations of Lyme borreliosis. J. Clin. Psychiatry 54, 263–268. 8335653

[B24] Fernández-Real J.-M. López-Bermejo A. Vendrell J. Ferri M. J. Recasens M. Ricart W. (2006). Burden of infection and insulin resistance in healthy middle-aged men. Diabetes Care 29, 1058–1064. 10.2337/diacare.2951058 16644637

[B25] Franceschi C. Bonafè M. Valensin S. Olivieri F. De Luca M. Ottaviani E.. (2000). Inflammaging: an evolutionary perspective on immunosenescence. Ann. N Y Acad. Sci. 908, 244–254. 10.1111/j.1749-6632.2000.tb06651.x 10911963

[B26] Gajewski P. D. Falkenstein M. Hengstler J. G. Golka K. (2014). *Toxoplasma gondii* impairs memory in infected seniors. Brain Behav. Immun. 36, 193–199. 10.1016/j.bbi.2013.11.019 24321215

[B27] Galloway P. H. Warner S. J. Morshed M. G. Mikelberg F. S. (2003). *Helicobacter pylori* infection and the risk for open-angle glaucoma. Ophthalmology 110, 922–925. 10.1016/s0161-6420(03)00093-9 12750090

[B28] Garakani A. Mitton A. G. (2015). New-onset panic, depression with suicidal thoughts and somatic symptoms in a patient with a history of Lyme disease. Case Rep. Psychiatry 2015:457947. 10.1155/2015/457947 25922779 PMC4397473

[B29] Garko M. G. (2012). The terrain within: a naturalistic way to think about and practice good health and wellness. Health and Wellness Monthly. Available online at: http://www.letstalknutrition.com [Accessed on May 26, 2018].

[B30] Gérard H. C. Dreses-Werringloer U. Wildt K. S. Deka S. Oszust C. Balin B. J.. (2006). *Chlamydophila (Chlamydia) pneumoniae* in the Alzheimer’s brain. FEMS Immunol. Med. Microbiol. 48, 355–366. 10.1111/j.1574-695X.2006.00154.x 17052268

[B31] Gilsanz P. Mayeda E. R. Glymour M. Quesenberry C. P. Mungas D. DeCarli C. S. (2017). Birth in a high infant mortality state: race and risk of dementia. Alzheimers Dement. 13, P210–P211. 10.1016/j.jalz.2017.07.082

[B32] Gray A. V. Michels K. S. Lauer A. K. Samples J. R. (2004). *Bartonella henselae* infection associated with neuroretinitis, central retinal artery and vein occlusion, neovascular glaucoma, and severe vision loss. Am. J. Ophthalmol. 137, 187–189. 10.1016/s0002-9394(03)00784-0 14700670

[B33] Greene J. A. Keohen G. F. (1943). Insulin resistance due to infection in diabetes mellitus in man. J. Am. Med. Assoc. 121, 173–176. 10.1001/jama.1943.02840030011003

[B34] Haas P. Steindl K. Schmid-Kubista K. E. Aggermann T. Krugluger W. Hageman G. S.. (2009). Complement factor H gene polymorphisms and *Chlamydia pneumoniae* infection in age-related macular degeneration. Eye 23, 2228–2232. 10.1038/eye.2008.422 19169230 PMC4853919

[B35] Han Y. W. Fardini Y. Chen C. Iacampo K. G. Peraino V. A. Shamonki J. M.. (2010). Term stillbirth caused by oral *Fusobacterium nucleatum*. Obstet. Gynecol. 115, 442–445. 10.1097/AOG.0b013e3181cb9955 20093874 PMC3004155

[B36] Hazlett L. D. Stein-Streilein J. (Ed.) (2012). “The eye as a model for immune privilege,” in Infection, Immune Homeostasis and Immune Privilege, (Basel: Springer), 1–29. 10.1007/978-3-0348-0445-5

[B37] Holmes C. Cunningham C. Zotova E. Woolford J. Dean C. Kerr ua S.. (2009). Systemic inflammation and disease progression in Alzheimer disease. Neurology 73, 768–774. 10.1212/WNL.0b013e3181b6bb95 19738171 PMC2848584

[B38] House P. (2018). Edmond halley mortality rates, Diagram. [online] Pierre-marteau.com. Available online at: http://www.pierre-marteau.com/editions/1693-mortality/halley-mortality-4.html [Accessed on 13 February 2018].

[B39] Jeffcoat M. K. Geurs N. C. Reddy M. S. Cliver S. P. Goldenberg R. L. Hauth J. C. (2002). Periodontal infection and preterm birth: results of a prospective study. Obstet. Gynecol. Surv. 57, 5–6. 10.1097/00006254-200201000-00003 11480640

[B40] Johnson L. A. Sohrabi H. R. Hall J. R. Kevin T. Edwards M. O’Bryant S. E.. (2015). A depressive endophenotype of poorer cognition among cognitively healthy community—dwelling adults: results from the Western Australia memory study. Int. J. Geriatr. Psychiatry 30, 881–886. 10.1002/gps.4231 25394326

[B41] Jones J. L. Parise M. E. Fiore A. E. (2014). Neglected parasitic infections in the United States: toxoplasmosis. Am. J. Trop. Med. Hyg. 90, 794–799. 10.4269/ajtmh.13-0722 24808246 PMC4015566

[B42] Jung B.-K. Pyo K.-H. Shin K. S. Hwang Y. S. Lim H. Lee S. J.. (2012). *Toxoplasma gondii* infection in the brain inhibits neuronal degeneration and learning and memory impairments in a murine model of Alzheimer’s disease. PLoS One 7:e33312. 10.1371/journal.pone.0033312 22470449 PMC3310043

[B43] Kaarniranta K. Salminen A. Haapasalo A. Soininen H. Hiltunen M. (2011). Age-related macular degeneration (AMD): Alzheimer’s disease in the eye? J. Alzheimers Dis. 24, 615–631. 10.3233/JAD-2011-101908 21297256

[B44] Kawai U. Uchida N. Oono M. Fujita-Nakata M. Nakanishi M. Sanada M. (2018). Relationship of systemic inflammation and cellular immunity with advancement of cognitive decline in patients with Alzheimer’s disease. Clin. Exp. Neuroimmunol. [Epub ahead of print]. 10.1111/cen3.12454

[B45] Kellam P. Weiss R. A. (2006). Infectogenomics: insights from the host genome into infectious diseases. Cell 124, 695–697. 10.1016/j.cell.2006.02.003 16497580 PMC7119327

[B46] Kemp M. W. Musk G. C. Usuda H. Saito M. (2017). “Infection-associated preterm birth: advances from the use of animal models,” in Animal Models for the Study of Human Disease, 2nd Edn, ed. Michael Conn P. (Cambridge, MA: Academic Press), 769–804.

[B47] Kravetz J. D. Federman D. G. (2005). Toxoplasmosis in pregnancy. Am. J. Med. 118, 212–216. 10.1016/j.amjmed.2004.08.023 15745715

[B50] Lin W.-R. Wozniak M. A. Wilcock G. K. Itzhaki R. F. (2002). Cytomegalovirus is present in a very high proportion of brains from vascular dementia patients. Neurobiol. Dis. 9, 82–87. 10.1006/nbdi.2001.0465 11848687

[B51] London A. Benhar I. Schwartz M. (2013). The retina as a window to the brain-from eye research to CNS disorders. Nat. Rev. Neurol. 9, 44–53. 10.1038/nrneurol.2012.227 23165340

[B52] Luciano R. Barraco G. M. Muraca M. Ottino S. Spreghini Rita M. Sforza R. W.. (2015). Biomarkers of Alzheimer disease, insulin resistance and obesity in childhood. Pediatrics 135, 1074–1081. 10.1542/peds.2014-2391 25963004

[B53] Maahs D. M. West N. A. Lawrence J. M. Mayer-Davis E. J. (2010). Epidemiology of type 1 diabetes. Endocrinol. Metab. Clin. North Am. 39, 481–497. 10.1016/j.ecl.2010.05.011 20723815 PMC2925303

[B54] Malaguarnera M. Bella R. Alagona G. Ferri R. Carnemolla A. Pennisi G. (2004). *Helicobacter pylori* and Alzheimer’s disease: a possible link. Eur. J. Intern. Med. 15, 381–386. 10.1016/j.ejim.2004.05.008 15522573

[B55] Marcus M. (2016). Kris Kristofferson’s Lyme disease misdiagnosed as Alzheimer’s. CBS News. Available online at: https://www.cbsnews.com/news/kris-kristofferson-misdiagnosed-alzheimers-has-lyme-disease/ [Accessed on June 11, 2018].

[B56] Marquand R. Muller R. (1997). *Borrelia burgdorferi*: a risk factor for Alzheimer’s disease. Eur. Psychiatry 12:213s 10.1016/s0924-9338(97)80664-5

[B57] Mayeda E. R. Whitmer R. A. Yaffe K. (2015). Diabetes and cognition. Clin. Geriatr. Med. 31, 101–115. 10.1016/j.cger.2014.08.021 25453304 PMC4370221

[B58] McKinnon S. J. (2003). Glaucoma: ocular Alzheimer’s disease. Front. Biosci. 8, s1140–s1156. 10.2741/1172 12957857

[B59] Miklossy J. (1994). “Alzheimer disease—a spirochetosis?” in Alzheimer Disease, eds Giacobini E. Becker R. E. (Boston, MA: Birkhäuser), 41–45.

[B62] Miklossy J. (2011). Alzheimer’s disease-a neurospirochetosis. Analysis of the evidence following Koch’s and Hill’s criteria. J. Neuroinflammation 8:90. 10.1186/1742-2094-8-90 21816039 PMC3171359

[B60] Miklossy J. Kis A. Radenovic A. Miller L. Forro L. Martins R.. (2006). β-amyloid deposition and Alzheimer’s type changes induced by Borrelia spirochetes. Neurobiol. Aging 27, 228–236. 10.1016/j.neurobiolaging.2005.01.018 15894409

[B61] Miklossy J. Khalili K. Gern L. Ericson R. L. Darekar P. Bolle L.. (2004). *Borrelia burgdorferi* persists in the brain in chronic lyme neuroborreliosis and may be associated with Alzheimer disease. J. Alzheimers Dis. 6, 639–649. 10.3233/JAD-2004-6608 15665404

[B49] Montoya J. G. Liesenfeld O. (2004). Toxoplasmosis. Lancet 363, 1965–1976. 10.1016/S0140-6736(04)16412-X 15194258

[B63] Morrison R. E. Lancaster L. Lancaster D. J. Land M. A. (1991). Rocky mountain spotted fever in the elderly. J. Am. Geriatr. Soc. 39, 205–208. 10.1111/j.1532-5415.1991.tb01628.x 1991953

[B64] Mortimer J. A. Borenstein A. R. Gosche K. M. Snowdon D. A. (2005). Very early detection of Alzheimer neuropathology and the role of brain reserve in modifying its clinical expression. J. Geriatr. Psychiatry Neurol. 18, 218–223. 10.1177/0891988705281869 16306243 PMC1405917

[B65] Nash R. W. Lindquist T. D. (1992). Bilateral angle-closure glaucoma associated with uveal effusion: presenting sign of HIV infection. Surv. Ophthalmol. 36, 255–258. 10.1016/0039-6257(92)90094-a 1549809

[B66] Nibali L. Henderson B. Sadiq S. T. Donos N. (2014). Genetic dysbiosis: the role of microbial insults in chronic inflammatory diseases. J. Oral Microbiol. 6:22962. 10.3402/jom.v6.22962 24578801 PMC3936111

[B67] Offenbacher S. Katz V. Fertik G. Collins J. Boyd D. Maynor G.. (1996). Periodontal infection as a possible risk factor for preterm low birth weight. J. Periodontol. 67, 1103–1113. 10.1902/jop.1996.67.10s.1103 8910829

[B68] Ohno-Matsui K. (2011). Parallel findings in age-related macular degeneration and Alzheimer’s disease. Prog. Retin. Eye Res. 30, 217–238. 10.1016/j.preteyeres.2011.02.004 21440663

[B69] Olfson M. Kroenke K. Wang S. Blanco C. (2014). Trends in office-based mental health care provided by psychiatrists and primary care physicians. J. Clin. Psychiatry 75, 247–253. 10.4088/jcp.13m08834 24717378

[B70] Opie E. L. (1929). Inflammation and immunity. J. Immunol. 17, 329–342.

[B71] Ott A. Stolk R. P. van Harskamp F. Pols H. A. P. Hofman A. Breteler M. M. B. (1999). Diabetes mellitus and the risk of dementia The Rotterdam Study. Neurology 53, 1937–1937. 10.1212/WNL.53.9.1937 10599761

[B72] Peterson-Kaiser Health System Tracker (2018). How much does the U.S. spend to treat different diseases?—Peterson-Kaiser Health System Tracker. Available online at: https://www.healthsystemtracker.org/chart-collection/much-u-s-spend-treat-different-diseases/?_sf_s=disease#item-circulatory-ill-defined-conditions-check-ups-largest-category-spending [Accessed on 13 February 2018].

[B73] Rashno M. M. Fallahi S. Bahrami P. (2017). Alzheimer’s disease and *Toxoplasma gondii* infection; seromolecular assess the possible link among patients. Int. J. Geriatr. Psychiatry 32, 232–234. 10.1002/gps.4616 28093864

[B74] Rashno M. M. Fallahi S. Kheirandish F. Bagheri S. Kayedi Hassan M. Birjandi M. (2016). Seroprevalence of *Toxoplasma gondii* infection in patients with Alzheimer’s disease. Arch. Clin. Infect. Dis. 11:e60133 10.5812/archcid.37205

[B75] Ravnskov U. McCully K. S. (2009). Vulnerable plaque formation from obstruction of vasa vasorum by homocysteinylated and oxidized lipoprotein aggregates complexed with microbial remnants and LDL autoantibodies. Ann. Clin. Lab. Sci. 39, 3–16. 19201735

[B76] Rebman A. W. Bechtold K. T. Yang T. Mihm E. A. Soloski M. J. Novak C.. (2017). The clinical, symptom, and quality of life characterization of a well-defined group of patients with post-treatment Lyme disease syndrome. Front. Med. 4:224. 10.3389/fmed.2017.00224 29312942 PMC5735370

[B77] Rivas A. L. Leitner G. Jankowski M. D. Hoogesteijn A. L. Iandiorio M. J. Chatzipanagiotou S.. (2017). Nature and consequences of biological reductionism for the immunological study of infectious diseases. Front. Immunol. 8:612. 10.3389/fimmu.2017.00612 28620378 PMC5449438

[B78] Riviere G. R. Riviere K. H. Smith K. S. (2002). Molecular and immunological evidence of oral Treponema in the human brain and their association with Alzheimer’s disease. Oral Microbiol. Immunol. 17, 113–118. 10.1046/j.0902-0055.2001.00100.x 11929559

[B79] Rizzo A. Paolillo R. Iafusco D. Prisco F. Romano Carratelli C. (2012). *Chlamydia pneumoniae* infection in adolescents with type 1 diabetes mellitus. J. Med. Microbiol. 61, 1584–1590. 10.1099/jmm.0.048512-0 22859582

[B80] Rock K. L. Kono H. (2008). The inflammatory response to cell death. Annu. Rev. Pathol. 3, 99–126. 10.1146/annurev.pathmechdis.3.121806.151456 18039143 PMC3094097

[B81] Rodriguez A. R. Plascencia-Villa G. Witt C. M. Yu J.-J. José-Yacamán M. Chambers J. P.. (2015). Chlamydia pneumoniae promotes dysfunction of pancreatic β cells. Cell. Immunol. 295, 83–91. 10.1016/j.cellimm.2015.03.010 25863744 PMC4533996

[B82] Rogers J. (1995). Inflammation as a pathogenic mechanism in Alzheimer’s disease. Arzneimittelforschung 45, 439–442. 7763341

[B83] Saczynski J. S. Beiser A. Seshadri S. Auerbach S. Wolf P. A. Au R. (2010). Depressive symptoms and risk of dementia: the Framingham Heart study. Neurology 75, 35–41. 10.1212/WNL.0b013e3181e62138 20603483 PMC2906404

[B84] Schulz P. Spannhorst S. Iffland B. Toepper M. (2007). Alzheimer’s disease and *Toxoplasma gondii* infection; seromolecular assess the possible link among patients. Dement. Geriatr. Cogn. Disord. 17, 181–187.

[B86] Sekhsaria S. Rahbar F. Fomufod A. Mason R. Kosoko O. Trouth J. (1992). An unusual case of congenital cytomegalovirus infection with glaucoma and communicating hydrocephalus. Clin. Pediatr. 31, 505–507. 10.1177/000992289203100811 1322807

[B87] Sheets C. Grewal D. Greenfield D. S. (2009). Ocular toxoplasmosis presenting with focal retinal nerve fiber atrophy simulating glaucoma. J. Glaucoma 18, 129–131. 10.1097/IJG.0b013e318179f83f 19225349 PMC2702091

[B88] Solana R. Tarazona R. Gayoso I. Lesur O. Dupuis G. Fulop T. (2012). Innate immunosenescence: effect of aging on cells and receptors of the innate immune system in humans. Semin. Immunol. 24, 331–341. 10.1016/j.smim.2012.04.008 22560929

[B89] Soscia S. J. Kirby J. E. Washicosky K. J. Tucker S. M. Ingelsson M. Hyman B.. (2010). The Alzheimer’s disease-associated amyloid β-protein is an antimicrobial peptide. PLoS One 5:e9505. 10.1371/journal.pone.0009505 20209079 PMC2831066

[B90] Stafford R. S. Ausiello J. C. Misra B. Saglam D. (2000). National patterns of depression treatment in primary care. Prim. Care Companion J. Clin. Psychiatry 2, 211–216. 10.4088/pcc.v02n0603 15014631 PMC181143

[B91] Stagno S. Pass R. F. Cloud G. Britt W. J. Henderson R. E. Walton P. D. (1987). Primary cytomegalovirus infection in pregnancy: incidence, transmission to fetus, and clinical outcome. Obstet. Anesth. Digest. 7:46 10.1097/00132582-198707000-00007 3020264

[B92] Stoller K. P. (2015). Overview of Lyme disease: a critique of an ignored pandemic. Int. J. Curr. Adv. Res. 4, 409–414.

[B93] Stratton C. W. Wheldon D. B. (2006). Multiple sclerosis: an infectious syndrome involving *Chlamydophila pneumoniae*. Trends Microbiol. 14, 474–479. 10.1016/j.tim.2006.09.002 16996738

[B94] Su B. Wang X. Drew K. L. Perry G. Smith M. A. Zhu X. (2008). Physiological regulation of tau phosphorylation during hibernation. J. Neurochem. 105, 2098–2108. 10.1111/j.1471-4159.2008.05294.x 18284615 PMC3796382

[B96] Whitmer R. A. Biessels G. J. Quesenberry C. P. Liu J. Y. Karter A. J. Beeri M. (2015). Type 1 diabetes and risk of dementia in late life: the kaiser diabetes and cognitive aging study. Alzheimers Dement. 11, P179–P180. 10.1016/j.jalz.2015.07.147

[B97] Wilson R. S. Hoganson G. M. Rajan K. B. Barnes L. L. Mendes de Leon C. F. Evans D. A. (2010). Temporal course of depressive symptoms during the development of Alzheimer disease. Neurology 75, 21–26. 10.1212/WNL.0b013e3181e620c5 20603481 PMC2906401

[B98] Wong S.-Y. Remington J. S. (1994). Toxoplasmosis in pregnancy. Clin. Infect. Dis. 18, 853–861. 10.1093/clinids/18.6.853 8086543

[B99] World Health Organization (2011). The WHO STEP-Wise Approach Surveillance of Risk Factors For Non-Communicable Diseases. Geneva: World Health Organization.

[B100] Yuki K. Kimura I. Shiba D. Imamura Y. Tsubota K. (2010). Elevated serum immunoglobulin G titers against *Chlamydia pneumoniae* in primary open-angle glaucoma patients without systemic disease. J. Glaucoma 19, 535–539. 10.1097/IJG.0b013e3181ca7868 20164795

[B101] Zaidman G. W. (1997). The ocular manifestations of Lyme disease. Int. Ophthalmol. Clin. 37, 13–28. 10.1097/00004397-199703720-00003 9269595

